# 34.3 IMPROVING THE DETECTION OF INDIVIDUALS AT RISK OF DEVELOPING PSYCHOSIS IN PRIMARY MENTAL HEALTH CARE

**DOI:** 10.1093/schbul/sby014.144

**Published:** 2018-04-01

**Authors:** Jesus Perez, Huajie Jin, Debra A Russo, Jan Stochl, Michelle Painter, Sarah Byford, Peter Jones

**Affiliations:** 1 University of Cambridge; 2 King’s College London; 3 Cambridgeshire and Peterborough NHS Foundation Trust

## Abstract

**Background:**

General practitioners are usually the first health professionals contacted by people with early signs of psychosis. It is unclear whether increasing the intensity of liaison between primary care and secondary care improves the clinical effectiveness and cost-effectiveness of detecting people with, or at clinical high-risk (CHR-P) of developing, a first-episode psychosis (FEP). This is important given political commitments to facilitate early intervention and decrease waiting times in mental health. We developed and tested a theory-based intervention to improve detection and referral of these mental states.

**Methods:**

The LEGS study was a cluster randomised controlled trial (cRCT) involving primary care practices (clusters) in the county of Cambridgeshire and Peterborough, UK. Consenting practices were randomly allocated into two groups: (1) low-intensity liaison between primary care and secondary care, a postal campaign consisting of biannual guidelines to help in the identification and referral of individuals with early signs of psychosis and (2) the high-intensity intervention described in the previous section, which, in addition to the postal campaign, included a specialist mental health professional to liaise with each practice and support the theory-based educational package. Concealed randomisation involved a randomly permuted sequence in blocks, with 12 strata and 96 blocks. Practices that did not consent to be randomised constituted a practice-as-usual (PAU) group. The high- and low-intensity interventions were implemented over a period of 2 years for each practice during the study period April 2010 to October 2013.

The primary outcome was the number of CHR-P referrals to Early Intervention in Psychosis Services per practice site predicated on an assumption that the intensive intervention would double them. New referrals were assessed clinically and stratified into those who met criteria for CHR-P or FEP (together: psychosis true positives) and those who did not fulfil such criteria for psychosis (false positives). Referrals from PAU practices were also analysed.

An economic evaluation quantified the cost-effectiveness of the interventions and PAU, using decision-analytic modelling. Cost-effectiveness was expressed as the incremental cost per additional true positive identified.

**Results:**

Of the 104 eligible practices, 54 consented to be randomised. Twenty-eight practices were randomised to low-intensity liaison and 26 practices were randomised to the high-intensity liaison. Two high-intensity practices withdrew. High-intensity practices referred more CHR-P (incidence rate ratio (IRR) 2.2, 95% CI 0.9 to 5.1; p = 0.08)), FEP (IRR 1.9, 95% CI 1.05 to 3.4; p = 0.04) and true positive (IRR 2.0, 95% CI 1.1 to 3.6; p = 0.02) cases. High-intensity practices also referred more false positives (IRR 2.6, 95% CI 1.3 to 5.0; p = 0.005); most (68%) of these were referred on to appropriate services.

The total costs per true positive referral in high-intensity practices were lower than those in low-intensity or PAU practices; the high-intensity intervention was the most cost-effective strategy.

**Discussion:**

Increasing the resources aimed at managing the primary–secondary care interface provides clinical and economic value in this setting. Early detection of CHR-P in primary care is clinically and cost-effective

This talk will also introduce the continuation of this work; a 5-year research programme that will focus on the treatment of individuals with psychotic experiences in primary care settings.

